# Bi-Directional Full-Color Generation and Tri-Channel Information Encoding Based on a Plasmonic Metasurface

**DOI:** 10.3390/nano14131160

**Published:** 2024-07-07

**Authors:** Dewang Huo, Guoqiang Li

**Affiliations:** 1 Intelligent Optical Imaging and Sensing Group, Institute of Optoelectronics, State Key Laboratory of Photovoltaic Science and Technology, Shanghai Frontier Base of Intelligent Optoelectronics and Perception, Fudan University, Shanghai 200438, China; dwhuo@zhejianglab.com; 2 Zhejiang Laboratory, Hangzhou 311100, China

**Keywords:** plasmonic metasurface, full-color generation, tri-channel information encoding

## Abstract

Dynamic optical structural color is always desired in various display applications and usually involves active materials. Full-color generation, especially bi-directional full-color generation in both reflective and transmissive modes, without any active materials included, has rarely been investigated. Herein, we demonstrate a scheme of bi-directional full-color generation based on a plasmonic metasurface modulated by the rotation of the polarization angle of the incident light without varying the geometry and the optical properties of the materials and the environment where the metasurface resides. The metasurface unit cell consists of plasmonic modules aligning in three directions and is patterned in a square array. The metasurface structural color device is numerically confirmed to generate full colors in both reflection and transmission. Based on the proposed polarization-dependent structural color, the information encoding process is demonstrated for three multiplexed animal images and quick-responsive (QR) codes to verify the efficient information encoding and decoding of the proposed scheme. In the simulation, the animals can be seen under different polarization incidences, and the QR codes can be successfully decoded by the polarization rotation in transmission. The proposed bi-directional full-color generation metasurface has great potential in applications such as kaleidoscope generation, anti-counterfeiting, dynamic color display, and optical information encoding.

## 1. Introduction

Optical metasurfaces have drawn great attention in recent decades due to their distinctive properties, including perfect absorption [1,2], a negative refractive index [3], and phase engineering [4]. A large variety of metasurface devices have been demonstrated, such as perfect absorbers [5], color filters [6], and wavefront manipulators [7,8]. Due to their advantage of having a compact size, optical metasurface devices show great potential in applications for micro- and nano-optical systems [9]. In the field of color filters, traditional methods with pigments and dyes [10] suffer from fading, a low spatial resolution, and environmental pollution, which hinders their application in micro-optical systems or applications requiring a high spatial resolution. The structural color based on metasurfaces outperforms traditional dye and pigment coloration due to the advantages of non-fading, high spatial resolution, and environmental friendliness. In previous decades, structural color devices based on a multi-layer structure [11], dielectric nanostructure [12], and metallic nanostructure [6] have been extensively studied, and they were found to exhibit excellent color properties and a sub-wavelength spatial resolution. However, the response of the metasurface is usually fixed after being fabricated, which limits its potential to be applied to active optical systems. In order to make the metasurface dynamic, active materials are usually employed in the structures, such as electro-optical materials [13], electrochromic materials [14,15], phase-change materials [16,17], semiconductors [18], liquid crystals [19,20,21], electro-chemistry [22,23], and MEMS [24]. The underlying physics of the active material-based dynamic structural color is the strong dependence of the optical response of the nanostructures on the size parameters and the optical properties of the constituent structures and materials.

In addition to modulating the property of the constituent materials, tailoring the incidence or output light polarization also enables the anisotropic metasurfaces to be tuned. Most linear polarization angle tuning methods offer an orthogonal-polarization variation, enabling bi-functional tunability. This includes the generation of two colors and bi-information encoding [25,26,27,28]. Using an Al plasmonic metasurface with different sizes and periods in perpendicular directions, vivid colors and dark states were generated when the incidence polarization varied [25]. A plasmonic filter set with polarization-switchable color properties based upon arrays of asymmetric cross-shaped nanoapertures in an aluminum thin film was demonstrated, but it had a low transmission down to 10% [26]. Using an engineered plasmonic aperture with varying gaps, colors with high monochromaticity and a wide gamut were generated [27]. By leveraging dual-state plasmonic color filters, high-density micro-image encoding was demonstrated [28]. In another work, full-color generation was achieved with polarization-tunable perfect light absorption in an Al metal/insulator/metal structure by varying the size parameters [29]. Full-color generation in a metasurface realized by simply varying the polarization angle of the incident linearly polarized white light is rarely seen. Several studies have achieved rich color generation using apertures [30] and blocks [31] with long axes aligning in different directions. Metal/dielectric/metal structures have been proposed to generate vivid colors [32]. Polarization-controlled full-color tunable plasmonic metal/dielectric/metal pixels were reported consisting of three different types of color modules corresponding to three subtractive primary colors in reflection [33]. However, the simultaneous achievement of full-color generation in both reflection and transmission modes using the same structure has not yet been reported.

Here, we demonstrate, for the first time, bi-directional full-color generation using the same metasurface based on a simple polarization angle variation of the incident linearly polarized white light without any active materials involved. A full-color metasurface is obtained through the module design to incorporate multiple modules in the metasurface unit cell. In this metasurface, the modules in three directions are included, and as a result, optical modes corresponding to each module can be selectively excited by the linearly polarized light, and full color can be generated in both reflective and transmissive modes using the same structure. The potential of the proposed structural color filter method for encoding is demonstrated by integrating three pieces of optical information into one device. We designed three-channel information in one metasurface and successfully decoded the information on each channel by varying the polarization angle of the incident linearly polarized light in simulation. The proposed structural coloration method has great potential in dynamic color display, anti-counterfeiting, and optical information encoding.

## 2. Results and Discussions

**Figure 1:**
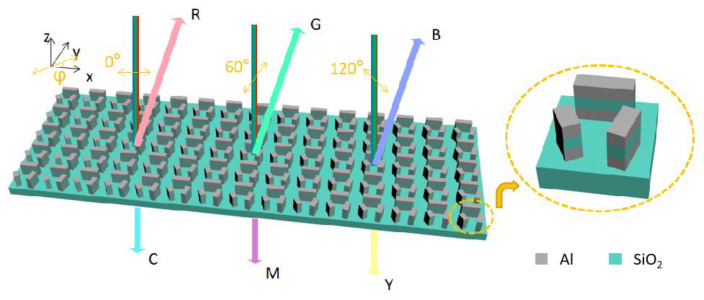
The schematics of the bi-directional full-color plasmonic metasurface and the enlarged plot of a unit cell in the metasurface.

In order to realize full-color generation by varying the linear polarization angle of the incident white light, a metasurface comprising three-module units was designed, as shown in Figure 1. To generate vivid colors, the plasmonic metal/insulator/metal structure was selected, and the Al was selected as the plasmonic material due to the excellent plasmonic property in the visible regime. The transparent SiO_2_ was adopted as the dielectric material. Other transparent dielectrics in the visible regime, such as Al_2_O_3_, may also be selected. Due to the polarization-dependent optical responses, the module exhibits polarization-dependent structural colors. The three modules are positioned in particular directions in the *x*-*y* plane, as shown in the enlarged plot of the unit cell in Figure 1. In both the reflection and transmission modes, various color generations can be achieved when the linearly polarized white light with varying polarization angles illuminates the metasurface, normally from above. The additive colors and the subtractive colors are generated in reflection and transmission, respectively.

**Figure 2:**
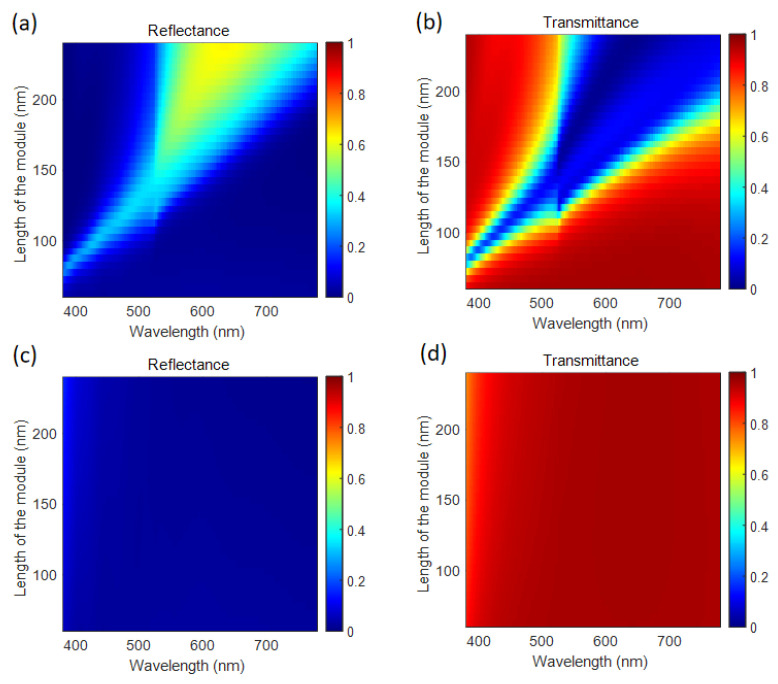
The spectra of the one-module metasurface versus the length of the Al/SiO_2_/Al module under the condition of (**a**,**b**) *x*-polarized incidence and (**c**,**d**) *y*-polarized incidence. (**a**,**c**) are the reflectance spectra; (**b**,**d**) are the transmittance spectra.

To evaluate the optical response of the plasmonic metasurface, the finite-difference time-domain method is adopted to perform the electromagnetic modeling of the metasurface, and the FDTD Solutions software (Ansys Lumerical 2022 R1.1) from Ansys Inc. (Canonsburg, PA, USA) is employed. For modeling, the periodic boundary conditions are applied along the *x*- and *y*-axes to simulate the square array of the metasurface while perfectly matched layer boundary conditions are established along the *z*-axis. The incident light normally illuminates the metasurface from above and propagates in the negative *z*-axis direction. The optical parameters of the materials, Al and SiO_2_, are available in ref. [34]. More details of the simulation can be found in the Supplementary Materials. First, the optical response of the metasurface with the unit cell consisting of one module is studied. The module is a rectangular post consisting of a three-layer structure (metal/dielectric/metal). Due to the outstanding plasmonic property in the visible regime, Al is selected as the plasmonic material. Transparent SiO_2_ is adopted as the dielectric layer and the substrate. The long axis of the module is positioned along the *x*-axis direction. The unit cells are distributed in a square array. The length of the module is changed to manipulate the optical response spectra when the period is set to 360 nm, the width of the module is 60 nm, and the thickness of each layer is 50 nm. Size-dependent spectra can be seen in both the reflection and transmission spectra in Figure 2. When the length of the module increases, the reflectance peak and the transmission dip redshift to the long wavelengths in the visible regime with the *x*-polarization white light incidence, as shown in Figure 2a,b. With the y-polarization white light incidence, there is no reflectance peak or transmission dip in the spectra where the reflectance is low throughout the visible regime to exhibit a ‘dark’ state in reflection, and the transmittance is high to exhibit a ‘bright’ state in transmission, as shown in Figure 2c,d. As such, the rectangular module acts as a polarization-dependent structural color filter in both the reflection and transmission modes. The peak and the dip positions in the spectra can be designed by changing the size of the module.

**Figure 3:**
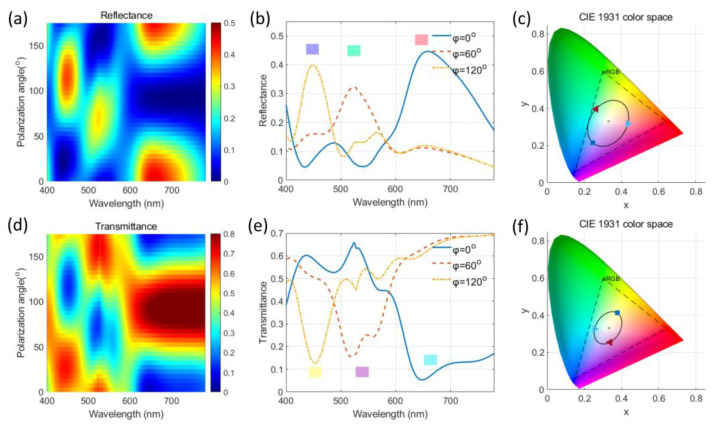
(**a**,**b**) The reflectance and (**d**,**e**) transmittance spectra of the plasmonic metasurface with respect to the varying incident linear polarization angle and (**c**,**f**) the plots of the CIE 1931 color space. The signs of the right triangle, left triangle, and square in (**c**,**f**) denote 0° polarization incidence, 60° polarization incidence, and 120° polarization incidence, respectively. The color bar in (**a**) denotes reflectance, and the color bar in (**d**) represents transmittance.

Since full color can be produced by mixing three primary colors, the three-module metasurface is designed to generate three primary colors when the incident light polarization is along the long axes of the three modules, respectively. In the metasurface shown in Figure 1, the three modules are positioned at 0°, 75°, and 120°, respectively, with respect to the *x*-axis direction for the consideration of mixing three primary colors. Each module consists of a three-layer structure of Al/SiO_2_/Al, and each layer has a thickness of 50 nm. The dimensions of the cross sections of the three modules are as follows: 210 nm × 60 nm for the 0°-aligned module, 125 nm × 70 nm for the 75°-aligned module, and 100 nm × 60 nm for the 120°-aligned module, respectively. The period of the metasurface is 360 nm. The dimensions of the modules are selected through optimization, aiming to generate full colors in both reflection and transmission with varying polarization angles of the incident light. Therefore, the modes corresponding to the long axis of the modules can be selectively excited by varying the linear polarization angle of the white light incidence. The reflectance spectra concerning the incident polarization angle are calculated and shown in Figure 3a. As expected, the three modes in the structure are, respectively, excited with respect to the polarization angle of incident linearly polarized light. As a result, there are three peaks at different wavelength ranges in the reflection spectra concerning varying polarization angles. In order to estimate the perceived color, the corresponding color is described in the CIE 1931 color space with a D65 standard illumination [23]. The CIE XYZ tristimulus values corresponding to the optical response spectra (reflection or transmission) are calculated as [35]

(1) $$X=\frac{1}{k}\int I\left(\lambda \right)S\left(\lambda \right)\bar{x}\left(\lambda \right)d\lambda$$

(2) $$Y=\frac{1}{k}\int I\left(\lambda \right)S\left(\lambda \right)\bar{y}\left(\lambda \right)d\lambda$$

(3) $$Z=\frac{1}{k}\int I\left(\lambda \right)S\left(\lambda \right)\bar{z}\left(\lambda \right)d\lambda$$

where *k* is a normalization factor, $I\left(\lambda \right)$ is the spectral energy distribution of the reference light, and $S\left(\lambda \right)$ is the far field reflectance or transmittance spectrum obtained from the designed metasurface under illumination. The $\bar{x}\left(\lambda \right)$, $\bar{y}\left(\lambda \right)$, and $\bar{z}\left(\lambda \right)$ are the CIE 1931 standard color-matching functions [35]. The chromaticity values *x* and *y* are then normalized as $x=X/\left(X+Y+Z\right)$ and $y=Y/\left(X+Y+Z\right)$, which fall between 0 and 1, to represent the colors in the CIE 1931 color space, as shown in Figure 3c,f, respectively.

The predicted color of a certain spectrum under illumination corresponds to a point in the CIE 1931 color space. As the polarization angle of the incident linearly polarized light varies, the color changes as well. The routine of the color variation of the metasurface circles the chromatic color point (denoted as the asterisk symbol shown in Figure 3c,f in the CIE 1931 color space, which indicates full-color generation from the metasurface. As the polarization angle varies from 0° to 180° counterclockwise, the color evolves from the red region to the green region and from the green region to the blue region, as seen in Figure 3c. As shown in Figure 3b, three reflectance spectra corresponding to the polarization angles of 0°, 60°, and 120° are plotted, where the three distinct peaks correspond to the red, green, and blue colors, respectively.

To evaluate the transmission performance of the metasurface, the transmittance spectra are also calculated, as depicted in Figure 3d. There are three transmission dips in the spectra with respect to the varying polarization angles of the incident linearly polarized white light. Contrary to the reflection case, the transmission dips in the spectra of the transmission case generate subtractive colors. In the CIE 1931 color space, the color transmitted from the device evolves from the cyan region to the magenta region and from the magenta region to the yellow region as the polarization angle of the incident linearly polarized light varies from 0° to 180° counterclockwise, as seen in Figure 3f. The transmitted colors circle the chromatic color point in the CIE 1931 color space. The transmission spectra corresponding to the polarization angles of 0°, 60°, and 120° are plotted in Figure 3e. There are three transmission dips located at different wavelengths corresponding to varying polarization angles of the linearly polarized incidence. To describe the perceived colors, they correspond to the cyan, magenta, and yellow colors, respectively. As a result, full subtractive colors can be generated from the metasurface in the transmission mode. In summary, full-color generation in both reflection and transmission can be successfully realized by the designed metasurface with respect to the varying polarization angle of the incident linearly polarized white light of D65 illumination. Additive full colors are generated in reflection, and subtractive full colors are generated in transmission.

**Figure 4:**
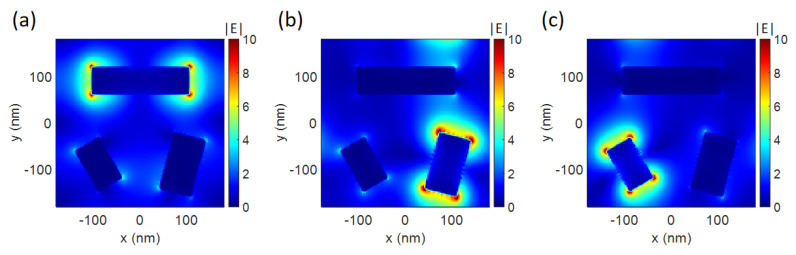
The electric field distribution in the *x*-*y* plane through centers of the upper Al posts in the metasurface under the conditions of (**a**) 0° incident polarization at the wavelength of 659.4 nm, (**b**) 60° incident polarization at the wavelength of 518.6 nm, and (**c**) 120° incident polarization at the wavelength of 444.2 nm. The color bars denote the magnitude of the electric field. The labels *x* and *y* denote the Cartesian coordinates in the *x*-*y* plane in Figure 1.

Bi-directional color generation by the metasurface originates from the optical properties of the modules. The in-phase plasmonic mode in the metal/dielectric/metal nanostructures can be excited to generate rich colors [36]. Due to the anisotropy of the module, the corresponding plasmonic mode can be excited by the linearly polarized light with polarization along the long axis of the module. When the incident linearly polarized light polarizes perpendicular to the long axis of the module, no resonance resides in the visible regime due to the small width of the module. To interpret the underlying physics of the polarization-dependent structural colors, the electromagnetic field in the metasurface is calculated. Due to the anisotropy of the modules, the resonance in each module can be selectively excited by the linearly polarized light. The electric field distributions of the nanostructures in the *x*-*y* plane, as the polarization angle varies, are illustrated in Figure 4a–c. The enhanced and localized electric field can be seen around the module when the incident light polarization is parallel to the orientation of the module at the plasmonic wavelength. When the incident polarization angle is 0°, the plasmonic resonance in the 0°-alignment module is excited at a wavelength of 659.4 nm. When the incident polarization angle is 60°, the plasmonic resonance in the 75°-alignment module is excited at a wavelength of 518.6 nm. And when the incident polarization angle is 120°, the plasmonic resonance in the 120°-alignment module is excited at a wavelength of 444.2 nm. Due to the plasmonic resonance in the modules, enhanced reflection and reduced transmission are generated at the plasmonic wavelengths. The tri-layer structure generates a peak in the reflection spectrum and a dip in the transmission spectrum when the incident linearly polarized light polarizes parallel to the long axis of a certain module. The polarization-dependent optical responses of the module enable the polarization-dependent structural colors to generate additive colors in reflection and subtractive colors in transmission, respectively. Based on the polarization-tunable optical response of the tri-layer module, polarization-tunable structural colors of interest can be customized by the module design method to choose different module sizes and orientations in the metasurface.

**Figure 5:**
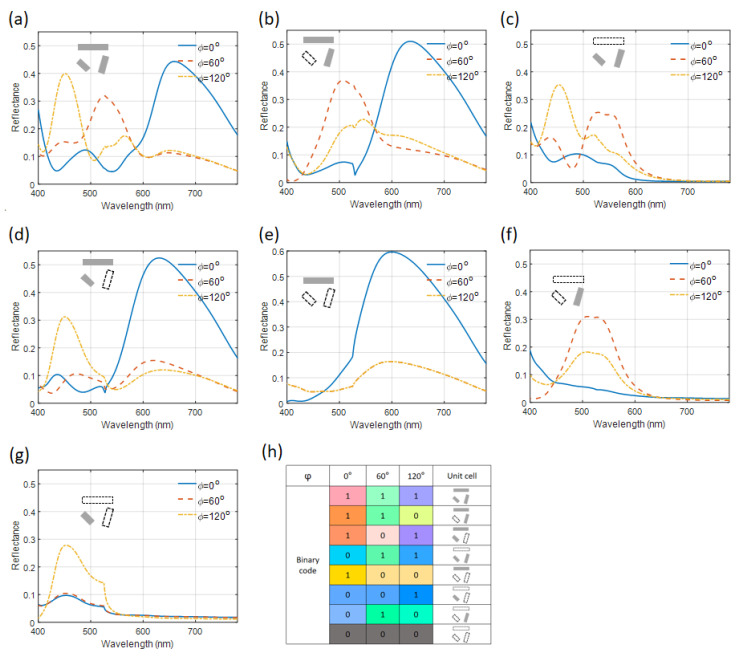
(**a**–**g**) The reflectance spectra of metasurfaces with different unit cells, as depicted inside the figures under the incidence polarization angles of 0°, 60°, and 120°. (**h**) A list of the binary code, the corresponding unit cells, and colors in reflection. The gray rectangles in the inserted schematics of (**a**–**h**) denote the existence of the Al/SiO_2_/Al module, and the rectangles outlined with dashed lines denote the absence of the Al/SiO_2_/Al module at the particular locations and with specific alignment directions.

Based on the polarization selectivity of the mode excitation of each module, the metasurface consisting of modules can be designed to manipulate the output. When three modules are included in the unit cell, the reflection spectra are acquired from simulations, and they are shown in Figure 5a. Three distinct reflection peaks are found at varying polarization angles of the incident light in the spectra. Altering the combinations of these modules within the unit cell changes the reflection spectra, as shown in Figure 5b–g. Notably, the center positions and the dimensions of each module, and the other settings in all simulations, remain the same as those in the three-module case. Initially, the setup transitions from a three-module to a two-module unit cell, affecting the reflectance spectra significantly. The corresponding reflectance spectra are shown in Figure 5b–d. When the two modules along the directions of 0° and 75° are utilized, reflectance peaks at the long wavelength side and in the range of 500~550 nm appear in the reflectance spectra with incidence polarizations of 0° and 60°, respectively. When the two modules along the directions of 75° and 120° are employed, reflectance peaks in the range of 500~550 nm and at the short wavelength side appear in the reflectance spectra with incidence polarizations of 60° and 120°, respectively. Similarly, when the two modules along the directions of 0° and 120° are used, reflectance peaks at the long wavelength side and short wavelength side appear in the reflectance spectra with incidence polarizations of 0° and 120°, respectively. Furthermore, when there is only one module in the unit cell, the reflectance peaks corresponding to the polarization angles of the incident light coinciding with the module directions show up, respectively, as shown in Figure 5e–g. In particular, the reflectance peak may also exist when the polarization angles are not in line with the module directions as a result of a non-orthogonal relation between the polarization angles and the module orientations. 

Some inspirations can be drawn from the above results. With the existence of the module in the 0° direction, there are reflectance peaks at the long wavelength side for all cases with the 0° polarization incidence, which leads to a similar color from red to yellow for the unit cells described in Figure 5. For unit cells containing the module in the 75° direction, there are peaks at the wavelength range from 500 nm to 550 nm with the 60° polarization incidence, which leads to a similar green color for unit cells. For unit cells containing the module in the 120° direction, there are peaks at the wavelength below 450 nm with the 120° polarization incidence, which leads to similar colors from blue to purple. These observations suggest that by adjusting the module configurations within a unit cell, it is possible to achieve a controlled reflection that can be used to encode information. This is carried out by altering the polarization angles of the incident linearly polarized white light and analyzing the resulting color in the reflection. The various configurations and their corresponding perceived colors are systematically cataloged in a look-up table, as shown in Figure 5h, to facilitate information encoding. In summary, by exploiting the polarization selectivity of each module, the metasurface enables precise control over the reflection properties of light, allowing for innovative ways to manipulate and encode optical information based on color perception under different light polarization conditions.

**Figure 6:**
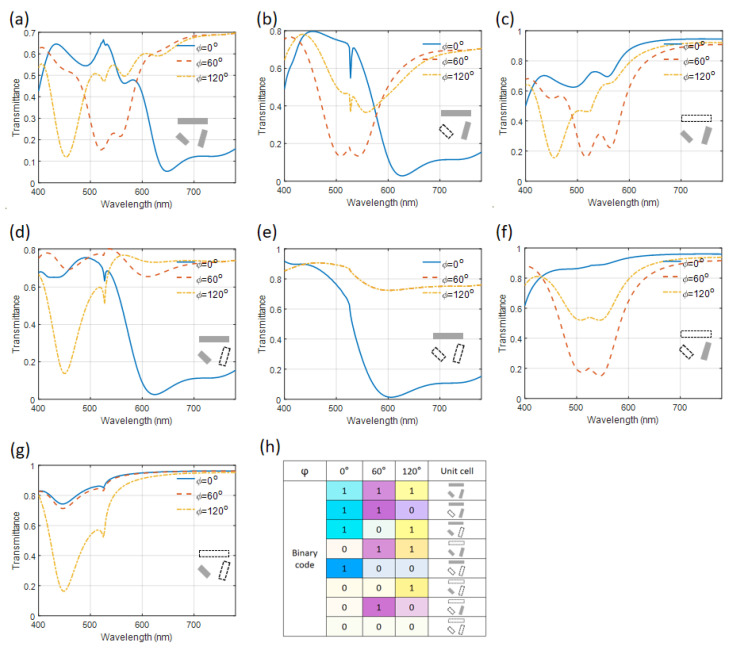
(**a**–**g**) The transmittance spectra of metasurfaces with different unit cells as depicted inside the figures under the incidence polarization angles of 0°, 60°, and 120°. (**h**) A list of the binary code, the corresponding unit cells, and colors in transmission. The gray rectangles in the inserted schematics of (**a**–**h**) denote the existence of the Al/SiO_2_/Al module, and the rectangles outlined with dashed lines denote the absence of the Al/SiO_2_/Al module at the particular locations and with specific alignment directions.

In the transmission configuration, the transmittance spectra of the various unit cell structures are meticulously detailed in Figure 6a–g, where subtractive structural colors are generated by the metasurface. With the existence of the module in the 0° direction in the metasurface, transmission dips are consistently observed at the long wavelength side across all scenarios involving 0° polarization incidence. This phenomenon consistently results in the generation of similar colors ranging from cyan to blue across the unit cells. Similarly, for unit cells that incorporate a module oriented at 75°, the transmission dips occur within a specific wavelength range from 500 nm to 550 nm when subjected to a 60° polarization incidence, culminating in a uniform magenta color across these unit cells. Furthermore, for the unit cells with the module in the 120° orientation, the transmission dips center around the 450 nm wavelength when exposed to the 120° polarization incidence, leading to a consistent yellow color output. The distinctive outputs of these structurally varied unit cells can be systematically distinguished by subjecting them to linearly polarized white light at varying polarization angles and subsequently analyzing the resultant color in transmission. Detailed listings of the binary code combinations, corresponding unit cell structures, and their respective transmission colors are provided in Figure 6h. Therefore, the existence of certain modules can be directly visualized by the perceived colors in transmission. The presented findings are compelling and indicate that the polarization-dependent binary information can be encoded to the plasmonic metasurface. This encoded information can be subsequently decoded by adjusting the polarization state of the incident light to analyze the resulting color transformations.

**Figure 7:**
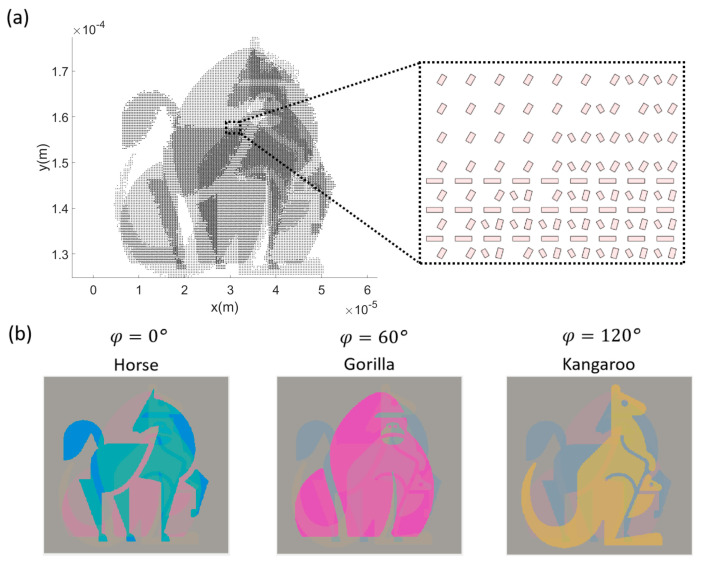
(**a**) The schematics of the designed 3-animal-images-in-1 metasurface. The enlarged plot outlines the detailed arrangement of the Al/SiO_2_/Al modules in the particular area. (**b**) The calculated optical images in the transmission of the designed 3-animal-images-in-1 metasurface with respect to different polarization angles of linear-polarized incident light.

The polarization-dependent color generation method can be adopted in the polarization-dependent information display, anti-counterfeiting, and optical information encoding in both reflection and transmission configurations. According to the polarization-dependent color modulation, different optical information can be encoded onto the metasurface corresponding to varying incident linearly polarized lights. Three pieces of binary information (e.g., pixels of 2D pictures) can be encoded into one unit cell, and the corresponding information can be decoded by tailoring the input linear polarization angle. Based on the look-up table in transmission, we first designed a metasurface with three information channels with respect to the incidence polarization angles of 0°, 60°, and 120°, each of which contains an image of a certain animal. As shown in Figure 7a, the metasurface is designed based on the look-up table in Figure 6h and the animal images in Figure S10a. For better performance, the alignment direction of the 75° module is modified to 60° except for the three-module unit cells. In the enlarged plot, we can see different unit cells at different locations corresponding to the encoding information. With the normal incidence of un-polarized white light illumination, the information of the three channels of animal images overlaps and cannot be distinguished. In contrast, with the linearly polarized white light illumination, the optical images in each channel can be obtained according to the table in Figure 6h and shown in Figure 7b. With different incident polarization angles, the main color of the optical image differs. With the 0° polarization incidence, the optical mode of the module along the 0° direction is excited and outperforms the other modes in the unit cell, so the perceived color of the corresponding unit cells is in the region of cyan, and the decoded image is an image of a horse in the colors of cyan and blue. Similarly, the unit cells with the module along the 75° or 60° direction under the 60° polarization incidence generate the color magenta, and the decoded image is an image of a gorilla in the color magenta. The unit cells, including the module along the 120° direction under the 120° polarization incidence, generate the color yellow, and the decoded image is an image of a kangaroo in the color yellow. As a result, three images of animals can be successfully encoded to one metasurface and decoded in transmission by varying the polarization angle of the linearly polarized incident white light.

**Figure 8:**
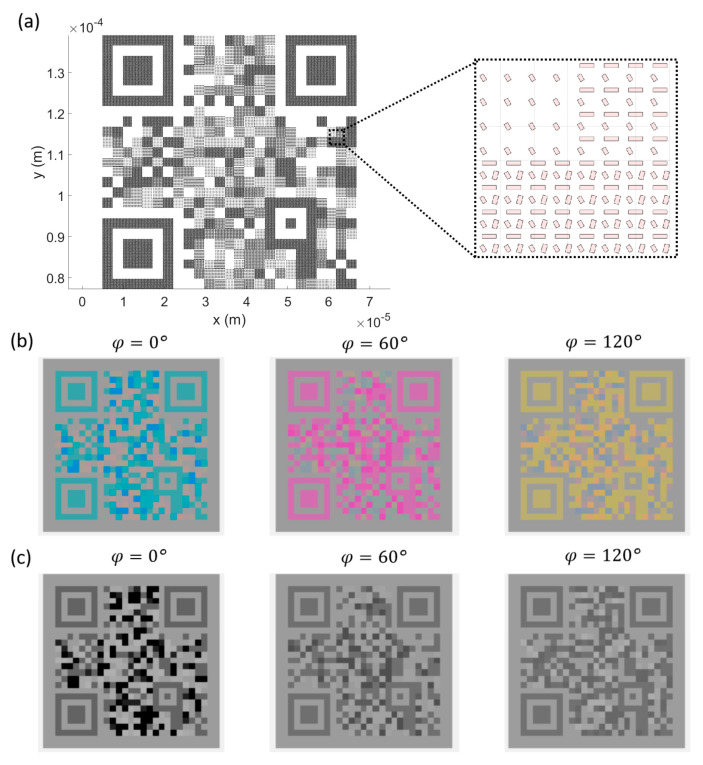
(**a**) The schematics of the designed 3-QR-codes-in-1 metasurface. The enlarged plot outlines the detailed arrangement of the Al/SiO_2_/Al modules in the particular area. (**b**) The calculated optical images in the transmission of the designed 3-QR-codes-in-1 metasurface with respect to different polarization angles of linear-polarized incident light. (**c**) The R component map of the RGB image under a 0° polarization incidence, the G component map of the RGB image under a 60° polarization incidence, and the B component map of the RGB image under a 120° polarization incidence.

As a second example, we designed a plasmonic metasurface containing three binary Quick Responsive (QR) Codes and examined them in a transmission configuration. The QR code is a two-dimensional binary information matrix and can include abundant information. The QR codes were designed to contain information of the ‘Zhejiang Lab’, ‘Metasurface’, and ‘Color Filter’, as shown in Figure S10b, respectively. Using the look-up table, multiple pieces of binary information can be encoded in the metasurface, as shown in the list in Figure 6h. The binary pixels of the QR codes corresponding to various unit cells were encoded into the nanostructures. The pattern is designed as shown in Figure 8a. Different unit cell structures can be seen at different locations in the metasurface due to the combination of three different binary information. To acquire the encoding information in the nanostructure pattern, the linearly polarized white light illuminates the pattern normally, and the transmission color is recorded to interpret the perceived colors. Upon different polarization angles of light, the colorful pattern in the transmission mode varies. Using the look-up table, the encoded binary information can be decoded from the colorful pattern. The colorful patterns are reproduced according to the table in Figure 6h and shown in Figure 8b. The QR codes can be successfully scanned to decode the containing information in the QR codes. Through the post-process of the colorful patterns, the QR codes can be reproduced more easily by plotting the R map of the RGB image under a 0° polarization incidence, the G component map of the RGB image under a 60° polarization incidence, and the B component map of the RGB image under a 120° polarization incidence, as shown in Figure 8c. The reproduced QR codes can be successfully scanned by a QR code scanning terminal, such as a smartphone, to decode the information as ‘Zhejiang Lab’, ‘Metasurface’, and ‘Color Filter’, respectively. According to the process described above, the optical encoding ability of the proposed metasurface is verified. Compared with other alternatives for all-optical encoding systems assisted by plasmonic effects, such as the encryption of photonic signals with an all-optical XOR logic gate [37], metasurface polarization information encoding has multiple advantages, such as increased security and complexity, a higher data density, less power consumption, and broader wavelength flexibility. And compared with other metasurface polarization encodings [27,28], the proposed metasurface can encode information with non-orthogonal polarizations, which significantly increases the information capacity of the metasurface.

In addition to the encoding application, the proposed polarization-tunable structural coloration has great potential in dynamic color display and anti-counterfeiting. Even though the proposed structure is deterministic, the proposed polarization information encoding mechanism can be designed to encode information into multiple modules to increase the decoding complexity in the anti-counterfeiting application. Full-color generation based on polarization tuning can also incorporate polarization rotation devices, such as liquid crystal optical devices, to form a fast-responsive dynamic structural color device. Moreover, the proposed structural color holds the potential to act as a polarization angle sensor to the linearly polarized light to display different colors in response to varying linear polarization angles.

## 3. Conclusions

In conclusion, bi-directional full-color generation from a plasmonic metasurface carried out by simply varying the incidence polarization angle was numerically proposed. A three-module metasurface was designed to show polarization-angle-selective optical responses so that the metasurface can be designed to realize different colors. By optimizing the size parameters of the metasurface, full color can be simultaneously generated in both reflection and transmission. The generated reflective color is an additive color, and the transmissive color is a subtractive color. We also explored the metasurface design with different module combinations in each unit cell. The module combination in the unit cell of the metasurface varied, and the optical response was systematically studied. The results show that the existence of a certain module can be visualized by the perceived color under linearly polarized white light illumination with a polarization parallel to the long axis of the module. Based on the polarization-tuning property of the metasurface, metasurfaces containing three-channel information were designed and tested with two examples of information from three animal images and information from three QR codes. The images of the animals were successfully regenerated, and the QR codes were successfully decoded to show the content encoded in the QR codes, which made information that the device can store more abundant. The proposed plasmonic structural color device shows great potential for applications in kaleidoscope generation, anti-counterfeiting, dynamic color display, and optical information encoding.

## Data Availability

The raw data supporting the conclusions of this article will be made available by the authors upon reasonable request.

## Supplementary Materials

The following supporting information can be downloaded at https://www.mdpi.com/article/10.3390/nano14131160/s1, Figure S1: The schematics of the proposed full-color metasurface in the FDTD simulation. Figure S2: The optical response of the Al/Al_2_O_3_/Al metasurface. Figure S3: The optical response of the Ag/SiO_2_/Ag metasurface. Figure S4: The schematics of the FDTD simulation region. Figure S5: The schematics of the metasurface design, collective transmission spectra, and isolated transmission spectra of the different unit cell sets. Figure S6: CIE 1931 chromaticity diagrams of the plasmonic metasurface with size deviations of −10%, −5%, −2%, 0%, 2%, 5%, and 10%. Figure S7: Optical information encoding considering a random ±2% size deviation. Figure S8: The optical response of the Al/SiO_2_/Al elliptical post metasurface. Figure S9: The absorption spectra of the proposed plasmonic metasurface concerning varying light polarizations. Figure S10: Target information images for encoding. Ref [34] is cited in the supplementary materials.

## References

[1] Landy N.I., Sajuyigbe S., Mock J.J., Smith D.R., Padilla W.J. (2008). Perfect Metamaterial Absorber. Phys. Rev. Lett..

[2] Hao J., Wang J., Liu X., Padilla W.J., Zhou L., Qiu M. (2010). High performance optical absorber based on a plasmonic metamaterial. Appl. Phys. Lett..

[3] Valentine J., Zhang S., Zentgraf T., Ulin-Avila E., Genov D.A., Bartal G., Zhang X. (2008). Three-dimensional optical metamaterial with a negative refractive index. Nature.

[4] Yu N., Genevet P., Kats M.A., Aieta F., Tetienne J.-P., Capasso F., Gaburro Z. (2011). Light propagation with phase discontinuities: Generalized laws of reflection and refraction. Science.

[5] Liu N., Mesch M., Weiss T., Hentschel M., Giessen H. (2010). Infrared Perfect Absorber and Its Application as Plasmonic Sensor. Nano Lett..

[6] Kumar K., Duan H., Hegde R.S., Koh S.C.W., Wei J.N., Yang J.K.W. (2012). Printing colour at the optical diffraction limit. Nat. Nanotechnol..

[7] Shelby R.A., Smith D.R., Schultz S. (2001). Experimental verification of a negative index of refraction. Science.

[8] Ni X., Ishii S., Kildishev A.V., Shalaev V.M. (2013). Ultra-thin, planar, Babinet-inverted plasmonic metalenses. Light Sci. Appl..

[9] Engheta N. (2007). Circuits with Light at Nanoscales: Optical Nanocircuits Inspired by Metamaterials. Science.

[10] Choi J., Kim S.H., Lee W., Yoon C., Kim J.P. (2012). Synthesis and characterization of thermally stable dyes with improved optical properties for dye-based LCD color filters. New J. Chem..

[11] Li Z., Butun S., Aydin K. (2015). Large-Area, Lithography-Free Super Absorbers and Color Filters at Visible Frequencies Using Ultrathin Metallic Films. ACS Photonics.

[12] Proust J., Bedu F., Gallas B., Ozerov I., Bonod N. (2016). All-Dielectric Colored Metasurfaces with Silicon Mie Resonators. ACS Nano.

[13] Guo J., Tu Y., Yang L., Zhang R., Wang L., Wang B. (2017). Electrically Tunable Gap Surface Plasmon-based Metasurface for Visible Light. Sci. Rep..

[14] Xiong K., Olsson O., Svirelis J., Palasingh C., Baumberg J., Dahlin A. (2021). Video Speed Switching of Plasmonic Structural Colors with High Contrast and Superior Lifetime. Adv. Mater..

[15] Gugole M., Olsson O., Rossi S., Jonsson M.P., Dahlin A. (2021). Electrochromic Inorganic Nanostructures with High Chromaticity and Superior Brightness. Nano Lett..

[16] Liu H., Dong W., Wang H., Lu L., Ruan Q., Tan You S., Simpson Robert E., Yang Joel K.W. (2020). Rewritable color nanoprints in antimony trisulfide films. Sci. Adv..

[17] Leitis A., Heßler A., Wahl S., Wuttig M., Taubner T., Tittl A., Altug H. (2020). All-Dielectric Programmable Huygens’ Metasurfaces. Adv. Funct. Mater..

[18] Wang W., Guan Z., Xu H. (2021). A high speed electrically switching reflective structural color display with large color gamut. Nanoscale.

[19] Franklin D., Chen Y., Vazquez-Guardado A., Modak S., Boroumand J., Xu D., Wu S.-T., Chanda D. (2015). Polarization-independent actively tunable colour generation on imprinted plasmonic surfaces. Nat. Commun..

[20] Franklin D., Frank R., Wu S.-T., Chanda D. (2017). Actively addressed single pixel full-colour plasmonic display. Nat. Commun..

[21] Shaltout A.M., Shalaev V.M., Brongersma M.L. (2019). Spatiotemporal light control with active metasurfaces. Science.

[22] Duan X., Liu N. (2018). Scanning Plasmonic Color Display. ACS Nano.

[23] Jia J., Ban Y., Liu K., Mao L., Su Y., Lian M., Cao T. (2021). Reconfigurable Full Color Display using Anisotropic Black Phosphorus. Adv. Opt. Mater..

[24] Holsteen A.L., Cihan A.F., Brongersma M.L. (2019). Temporal color mixing and dynamic beam shaping with silicon metasurfaces. Science.

[25] Olson J., Manjavacas A., Basu T., Huang D., Schlather A.E., Zheng B., Halas N.J., Nordlander P., Link S. (2016). High Chromaticity Aluminum Plasmonic Pixels for Active Liquid Crystal Displays. ACS Nano.

[26] Li Z., Clark A.W., Cooper J.M. (2016). Dual Color Plasmonic Pixels Create a Polarization Controlled Nano Color Palette. ACS Nano.

[27] Wen Y., Lin J., Chen K., Lin Y.S., Yang B.R. (2022). Full color metasurface with high-transmission and omnidirectional characteristics. Opt. Laser Technol..

[28] Heydari E., Sperling J.R., Neale S.L., Clark A.W. (2017). Plasmonic Color Filters as Dual-State Nanopixels for High-Density Microimage Encoding. Adv. Funct. Mater..

[29] Song M., Kudyshev Z.A., Yu H., Boltasseva A., Shalaev V.M., Kildishev A.V. (2019). Achieving full-color generation with polarization-tunable perfect light absorption. Opt. Mater. Express.

[30] Yun H., Lee S.-Y., Hong K., Yeom J., Lee B. (2015). Plasmonic cavity-apertures as dynamic pixels for the simultaneous control of colour and intensity. Nat. Commun..

[31] Kim M., Kim I., Jang J., Lee D., Nam K.T., Rho J. (2018). Active Color Control in a Metasurface by Polarization Rotation. Appl. Sci..

[32] Lio G.E., Ferraro A., Giocondo M., Caputo R., De Luca A. (2020). Color Gamut Behavior in Epsilon Near-Zero Nanocavities during Propagation of Gap Surface Plasmons. Adv. Opt. Mater..

[33] Feng R., Wang H., Cao Y., Zhang Y., Ng R.J.H., Tan Y.S., Sun F., Qiu C.-W., Yang J.K.W., Ding W. (2021). A Modular Design of Continuously Tunable Full Color Plasmonic Pixels with Broken Rotational Symmetry. Adv. Funct. Mater..

[34] Palik E.D. (1991). Handbook of Optical Constants of Solids II.

[35] Smith T., Guild J. (1931). The CIE colorimetric standards and their use. Trans. Opt. Soc..

[36] Wang H., Wang X., Yan C., Zhao H., Zhang J., Santschi C., Martin O.J.F. (2017). Full Color Generation Using Silver Tandem Nanodisks. ACS Nano.

[37] García-Beltrán G., Mercado-Zúñiga C., Torres-SanMiguel C.R., Gallegos-García G., Torres-Torres C. (2022). Photonic encryption by optical activity in Kerr-like carbon-based nanofluids with plasmonic nanoparticles. J. Mol. Liq..

