# Supplementary material for: Bi-Directional Full-Color Generation and Tri-Channel Information Encoding Based on a Plasmonic Metasurface
Source: Nanomaterials (Basel). 2024 Jul 7;14(13):1160. doi: 10.3390/nano14131160 (PMC11243537; doi:10.3390/nano14131160)
Supplement: Supplementary file 1 [file nanomaterials-14-01160-s001.zip › nanomaterials-2973686-supplementary.pdf]

nanomaterials

Supplementary materials

# Bi-directional full-color generation and tri-channel information encoding based on a plasmonic metasurface

Dewang Huo [1,2], Guoqiang Li [1,*]

1 Intelligent Optical Imaging and Sensing Group, Institute of Optoelectronics, State Key Laboratory of Photovoltaic Science and Technology, Shanghai Frontier Base of Intelligent Optoelectronics and Perception, Fudan University, Shanghai, 200438, China

2 Intelligent Optical Imaging and Sensing Group, Zhejiang Lab, Hangzhou, 311100, China

* Correspondence: gqli2001@gmail.com; gqli@fudan.edu.cn

## 1. The schematics of the proposed full-color metasurface and size parameters.

The software *FDTD Solutions* from *Ansys Inc.* was adopted to evaluate the optical response of the plasmonic metasurface. The schematics of the proposed full-color metasurface in the FDTD simulation are illustrated in Figure S1.

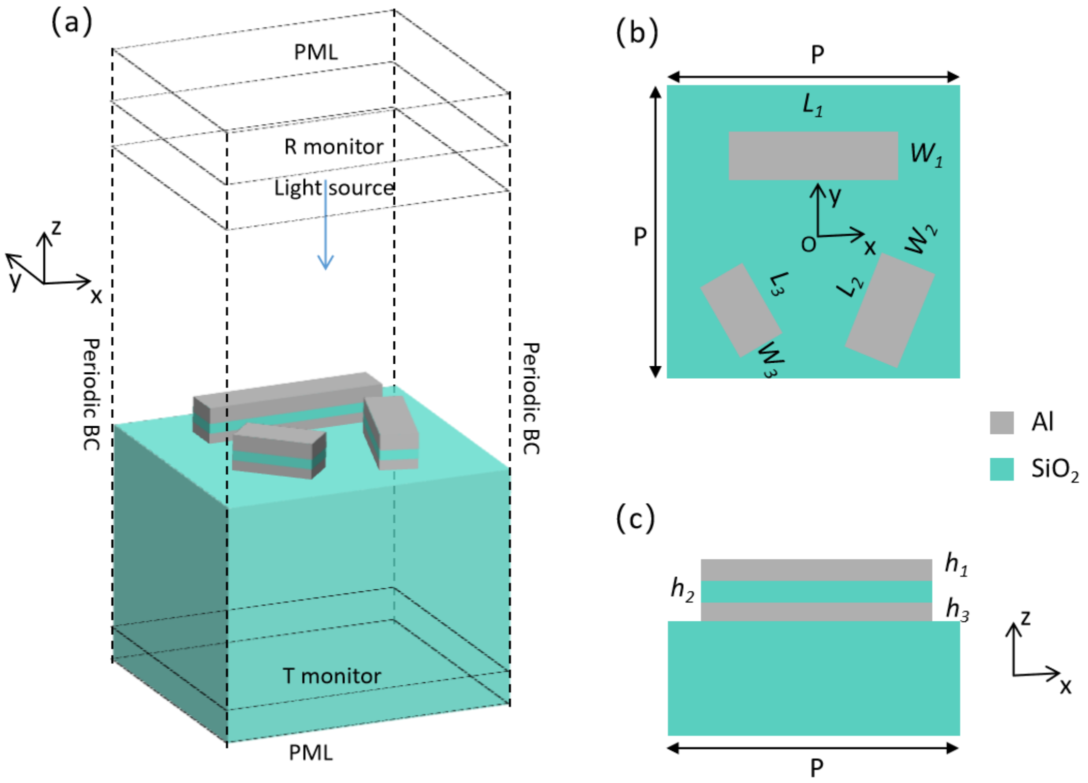


Figure S1. The schematics of the proposed full-color metasurface in the FDTD simulation. (a) The 3D view, (b) the top view, and (c) the front view.

There are three Al/$SiO_2$/Al modules on the $SiO_2$ substrate in the unit cell of the metasurface. The modules are located at coordinate positions (0,0.5P), (0.5P, -0.5P), and (-0.5P, -0.5P), respectively. The periodic boundary conditions are applied in the *x*- and *y*-axes to simulate the periodic array of the metasurface while the perfectly matched layer boundary conditions are established along the *z*-axis. The incident light is configured to illuminate the metasurface normally from above and propagates in the negative *z*-axis direction. The transmission and reflection are recorded using the field monitors. Optimization is carried out by varying the size and orientation parameters and the polarization angle of the

incident light, aiming to generate full colors in both reflection and transmission. The angle of the long axis of the module with respect to the $x$-axis is denoted as $\theta$. Finally, we select a set of parameters to demonstrate the bi-directional full-color generation of the metasurface in which $L_1$ = 210nm, $W_1$ = 60nm, $\theta_1$ = 0°, $L_2$ = 125nm, $W_2$ = 70nm, $\theta_2$ = 75°, $L_3$ = 100nm, $W_3$ = 60nm, $\theta_3$ = 120°, P = 360nm, and $h_1 = h_2 = h_3$ = 50nm. A mesh with dimensions of 2.5nm×2.5nm×2.5nm is used in the simulation. The optical parameters of the materials, Al and $SiO_2$, are available in ref. [1].

## 2. Possibility of selecting other constituent materials

We performed the simulation on the possible metasurface structural color by using $Al_2O_3$ instead of $SiO_2$ and Ag instead of Al, respectively, maintaining the same size parameters as the Al/$SiO_2$/Al case. The size parameters are as follows: $L_1$ = 210nm, $W_1$ = 60nm, $\theta_1$ = 0°, $L_2$ = 125nm, $W_2$ = 70nm, $\theta_2$ = 75°, $L_3$ = 100nm, $W_3$ = 60nm, $\theta_3$ = 120°, P = 360nm, and $h_1 = h_2 = h_3$ = 50nm. A mesh with dimensions of 2.5nm×2.5nm×2.5nm is used in the simulation. The optical parameters of the material $Al_2O_3$ can be found in ref. [1]. When using $Al_2O_3$ instead of $SiO_2$, the optical response of the metasurface exhibits a similar behavior with three main peaks in the reflection and three main dips in the transmission spectra, as shown in Figure S2. Due to the different refractive indices of the two materials, the structure needs to be optimized by further simulations. The results demonstrate that the $Al_2O_3$ material can also be adopted in the plasmonic structural color design.

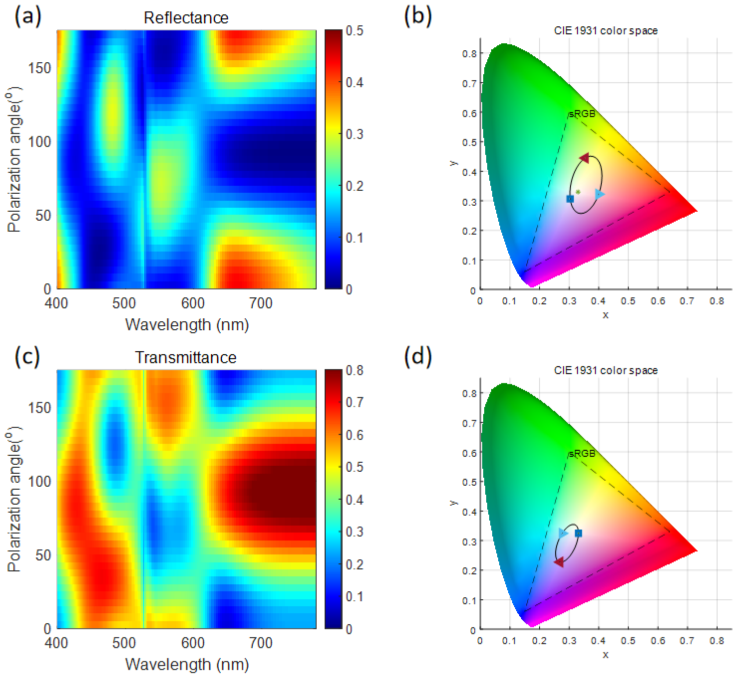


Figure S2. The optical response of the Al/$Al_2O_3$/Al metasurface in (a) reflection and (c) transmission, and the corresponding CIE 1931 chromaticity diagrams in (b) reflection and (d) transmission. The color bar in (a) denotes reflectance, and the color bar in (d) represents transmittance.

The reflection and transmission spectra obtained when using Ag instead of Al are detailed in Figure S3. The optical parameters of Ag can be found in ref. [1]. The size parameters are as follows: $L_1$ = 210nm, $W_1$ = 60nm, $\theta_1$ = 0°, $L_2$ = 125nm, $W_2$ = 70nm, $\theta_2$ = 75°, $L_3$ = 100nm, $W_3$ = 60nm, $\theta_3$ = 120°, P = 360nm, and $h_1 = h_2 = h_3$ = 50nm. A mesh with dimensions of 2.5nm×2.5nm×2.5nm is used in the simulation. The optical response of the Ag/$SiO_2$/Ag metasurface with the same geometric parameters differs from that of the proposed Al/$SiO_2$/Al metasurface. The Ag/$SiO_2$/Ag metasurface cannot produce full colors in this size configuration.

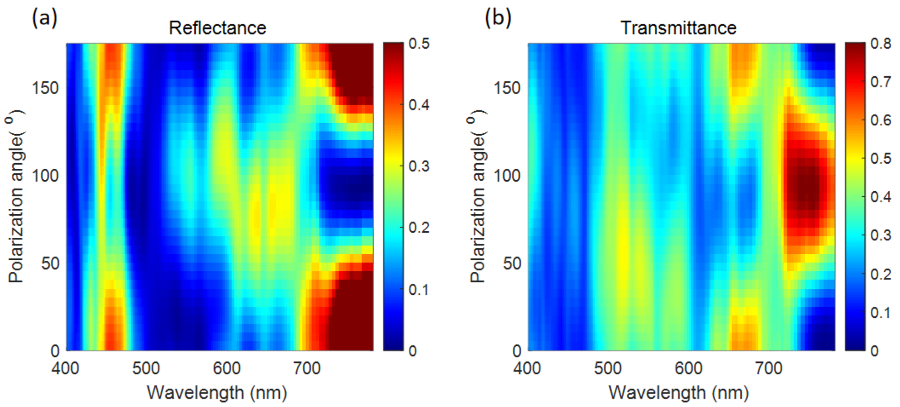


Figure S3. The optical response of the $Ag/SiO_2/Ag$ metasurface in (a) reflection and (b) transmission.

## 3. The numerical validation of the proposed encoding through FDTD modeling

The schematics of the FDTD modeling region are shown in Figure S4. The $SiO_2$ substrate beneath the nanostructures is omitted in the schematics. The PML boundary conditions are applied in the *z*-direction boundaries of the simulation region. The periodic boundary conditions are applied in the *x*- and *y*-direction boundaries of the simulation region. The incident light is configured to normally illuminate the metasurface from above and propagate along the negative *z*-axis direction. The size parameters of the modules are as follows: $L_1$ = 210nm, $W_1$ = 60nm, $\theta_1$ = 0°, $L_2$ = 125nm, $W_2$ = 70nm, $\theta_2$ = 75°, $L_3$ = 100nm, $W_3$ = 60nm, $\theta_3$ = 120°, P = 360nm, and $h_1 = h_2 = h_3$ = 50nm. The center positions of the modules are at coordinates (0,0.5P), (0.5P, -0.5P), and (-0.5P, -0.5P), respectively. A mesh with dimensions of 2.5nm×2.5nm×2.5nm is used in the simulation. The overall transmission spectrum is collected using a monitor covering the entire structure. The isolated spectra of the different sets are collected using four monitors near the metasurface covering the four different sets, respectively.

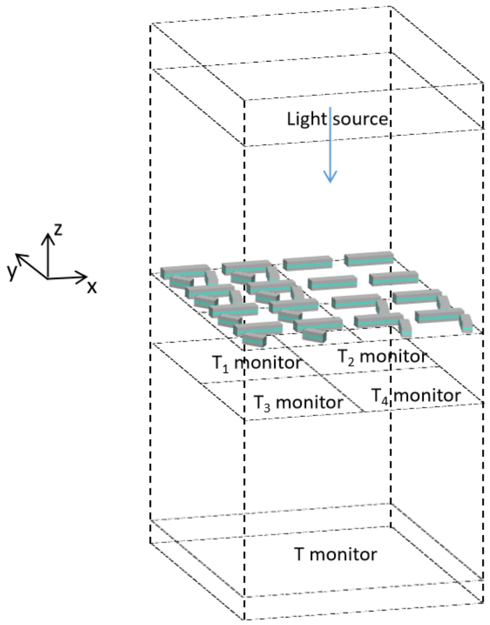


Figure S4. The schematics of the FDTD simulation region.

We designed a metasurface with four different unit cell sets, as shown in Figure S5(a). Each set consists of 2×2-unit cells. The gray rectangles in Figure S5(a) denote the existence of the $Al/SiO_2/Al$ module, and the rectangles outlined with dashed lines denote the absence of the $Al/SiO_2/Al$ module at the particular locations and alignment directions. The overall optical response and the isolated response of each set are analyzed. The overall optical response displays a

collective spectrum of all sets, as shown in Figure S5(b). The exact encoded pattern cannot be acquired from the collective spectra. The isolated optical response of each set shows the polarization-dependent response of the different unit cell structures, as detailed in Figure S5(c)-(f). The exact structure of the set can be obtained from the isolated spectra. For example, in Figure S5(d), the two transmission dips can be found at the long wavelength side with a 0° polarization incidence and in the range of 400nm-500nm with a 0° polarization incidence, respectively. The spectra indicate the existence of the modules aligned at 0° and 120°. The above results demonstrate that an encoded binary key can be distinguished from isolated spectra, it but cannot be distinguished from collective spectra.

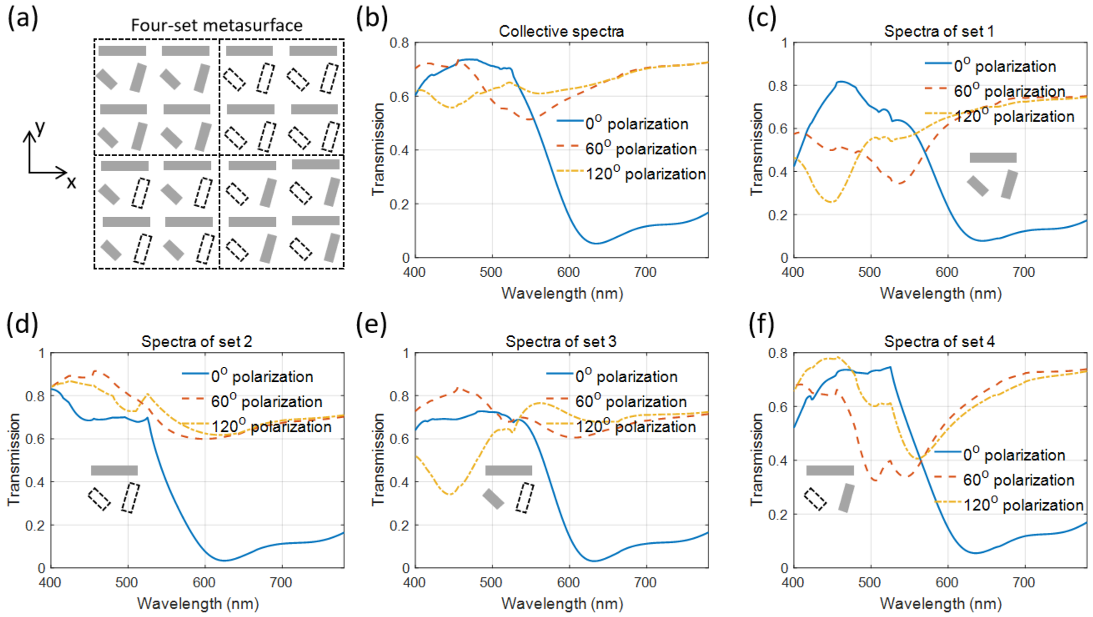


Figure S5. The schematics of the metasurface design, collective transmission spectra, and isolated transmission spectra of the different unit cell sets. The gray rectangles in the inserted schematics of (a) and (c-f) denote the existence of the Al/$SiO_2$/Al module, and the rectangles outlined with dashed lines denote the absence of the Al/$SiO_2$/Al module at the particular locations and with particular alignment directions.

## 4. Considering size deviation

We retested the bidirectional, full-color generation capabilities of the plasmonic metasurface by taking into account fabrication errors. We adjusted the error margins to -10%, -5%, -2%, 0%, 2%, 5%, and 10% of the lengths and widths of the modules. The period and the thickness of each layers are fixed at 360nm and 50nm, respectively. The colors corresponding to polarizations of 0°, 60°, and 120° are plotted in the CIE 1931 chromaticity diagrams in Figure S5. Due to the size-dependent property of the plasmonic resonances in the modules, the size deviation of the module length and width results in a color change in the metasurface in reflection and transmission, as depicted in Figure S6. With a ±10% size deviation, the produced colors largely deviate from those of the designed RGB or CMY targets (the '○' sign in the CIE 1931 chromaticity diagrams). With a ±2% size deviation, the produced colors remain similar to the designed RGB or CMY colors (the '○' sign in the CIE 1931 chromaticity diagrams). So, we state that the proposed metasurface demonstrates a size tolerance of about 2% to generate the RGB and CMY colors from the numerical results. In actual fabrication processes, the error depends on multiple parameters of the fabrication system. With optimized configurations of the fabrication system, the proposed metasurface can be precisely fabricated with a several-nanometer or even sub-nanometer precision.

To see the decoding performance of the proposed metasurface with a size deviation, the optical encoding examples in Figures 7 and 8 were recalculated using the data in Figure S6(b) and shown in Figure S7 while considering a random ± 2% size deviation. The information encoded into multiple channels can still be successfully decoded with the acceptable color deviation, as shown in the enlarged plot in Figure S6(c), where similar yellow colors are generated due to the small size deviation of the metasurface (±2%). The results indicate that the proposed metasurface encoding shows a good size tolerance of a ±2% size deviation.

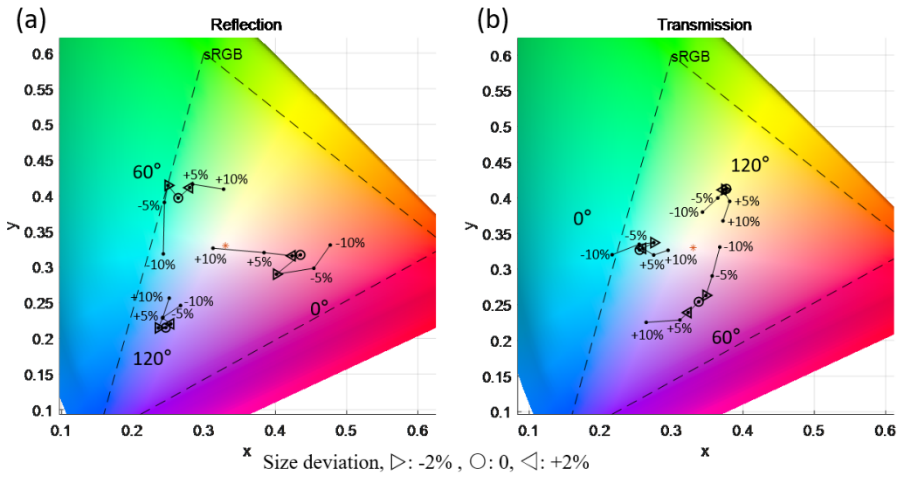


Figure S6. CIE 1931 chromaticity diagrams of the plasmonic metasurface with size deviations of -10%, -5%, -2%, 0%, 2%, 5%, and 10%. The color points are denoted with black dots.

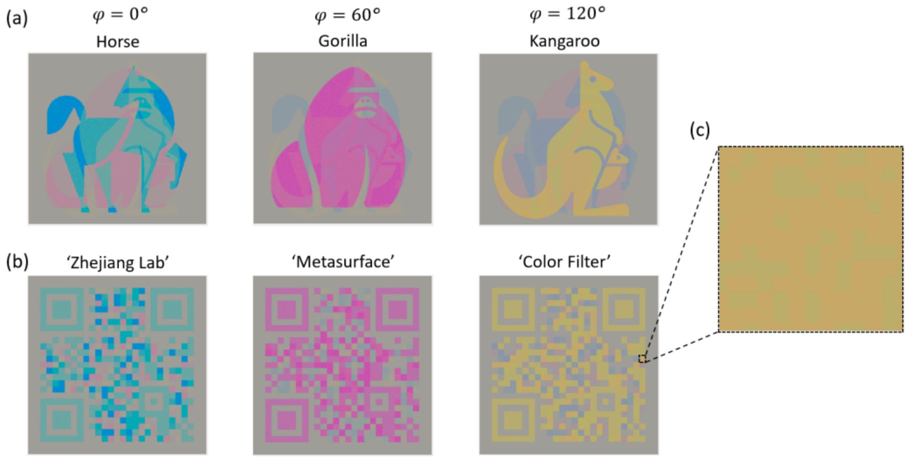


Figure S7. The calculated optical images in the transmission of the designed (a) 3-animal-images-in-1 metasurface and (b) 3-QR-codes-in-1 metasurface with respect to different polarization angles of linear-polarized incident light while considering a random ±2% size deviation. (c) An enlarged plot of a particular area in (b).

## 5. Considering other anisotropic structures

Except for the rectangular post used in the manuscript, other anisotropic structures such as elliptical posts may be also adopted. Similar to Figure S1, the rectangular posts are changed to elliptical posts. The center positions of the modules are still at coordinates (0,0.5P), (0.5P, -0.5P), and (-0.5P, -0.5P), respectively. The diameters of the long and short axes of

the elliptical modules are 220nm and 65nm for the 0°-aligned module, 155nm and 75nm for the 75°-aligned module, and 130nm and 65nm for the 120°-aligned module. The other parameters remain the same as those for the rectangular post metasurface. A mesh with dimensions of 2.5nm×2.5nm×2.5nm is used in the simulation. As shown in Figure S8, the simulation results show a similar optical response as that of the rectangular post metasurface. The optimized size parameters of the elliptical posts can be obtained through further simulations.

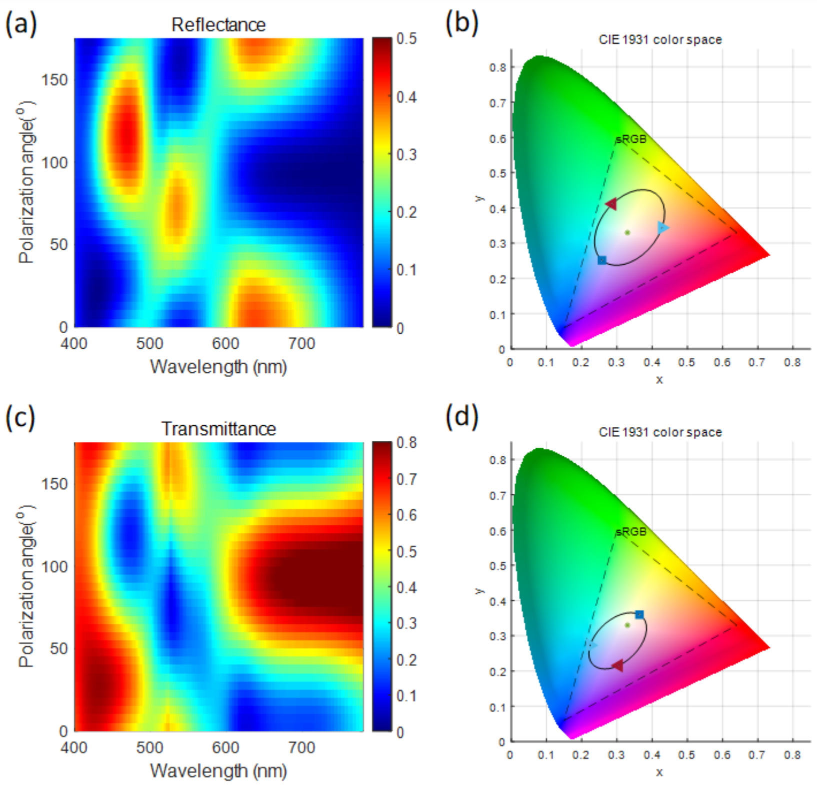


Figure S8. The optical response of the Al/$SiO_2$/Al elliptical post metasurface in (a) reflection and (c) transmission and the corresponding CIE 1931 chromaticity diagrams in (b) reflection and (d) transmission. The color bar in (a) denotes reflectance, and the color bar in (c) represents transmittance.

## 6. The absorption spectra of the metasurface

The absorption spectra concerning varying light polarization are calculated using the equation $A = 1 - R - T$ and are detailed in Figure S9. The absorption peak blueshifts when the incident light polarization angle varies from 0° to 180°, which is consistent with the polarization-dependent excitation of plasmonic resonances in the designed metasurface structural color.

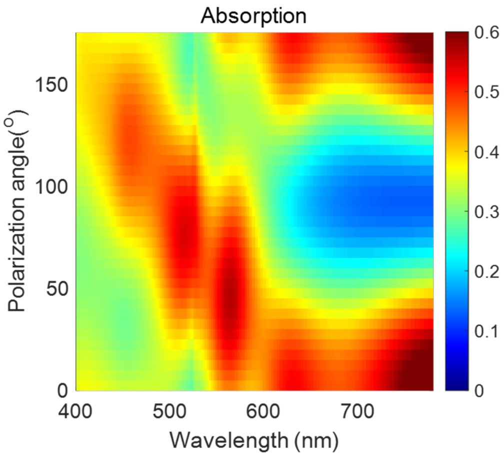


Figure S9. The absorption spectra of the proposed plasmonic metasurface concerning varying light polarizations. The color bar denotes absorption.

## 7. Targets for information encoding in Figures 7 and 8

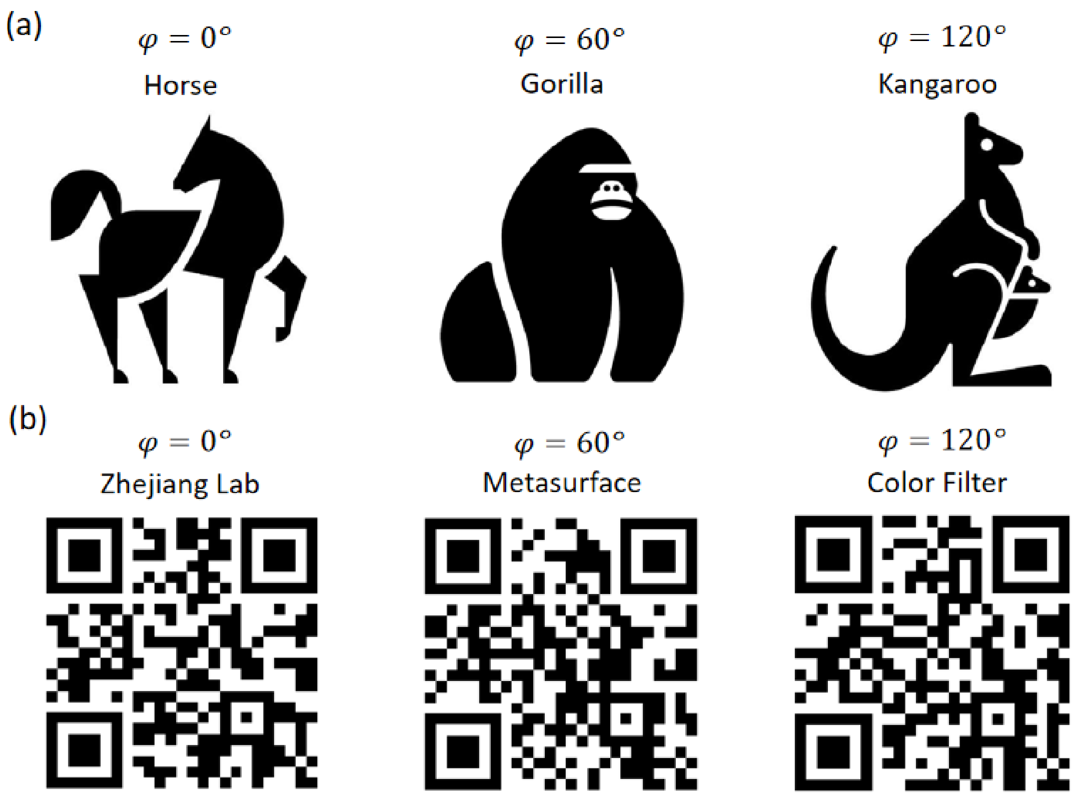


Figure S10. Target information images (a) for three-animal information encoding in Figure 7 and (b) for QR code encoding in Figure 8.

## Reference

1. Palik, E. D., *Handbook of Optical Constants of Solids II*. Boston Academic Press: Cambridge, MA, USA, 1991.
